# Laparoscopic Transabdominal Preperitoneal Inguinal Hernia Repair Using Memory-Ring Mesh: A Pilot Study

**DOI:** 10.1155/2016/9407357

**Published:** 2016-08-18

**Authors:** Takeshi Matsutani, Tsutomu Nomura, Nobutoshi Hagiwara, Akihisa Matsuda, Yoshimune Takao, Eiji Uchida

**Affiliations:** Department of Gastrointestinal Hepato-Biliary-Pancreatic Surgery, Nippon Medical School, Graduate School of Medicine, Tokyo 113-8603, Japan

## Abstract

*Purpose.* To evaluate the feasibility, safety, and effectiveness of laparoscopic transabdominal preperitoneal (TAPP) inguinal hernia repair using a memory-ring patch (Polysoft*™* mesh).* Patients and Methods.* Between April 2010 and March 2013, a total of 76 inguinal hernias underwent TAPP repair using Polysoft mesh in 67 adults under general anesthesia. Three different senior resident surgeons performed TAPP repair under the instruction of a specialist surgeon. Nine patients had bilateral hernias. The 76 hernias included 37 indirect inguinal hernias, 29 direct hernias, 1 femoral hernia, 1 pantaloon hernia (combined direct/indirect inguinal hernia), and 8 recurrent hernias after open anterior hernia repair. The immediate postoperative outcomes as well as the short-term outcomes (mainly recurrence and incidence of chronic pain) were studied.* Results.* There was no conversion from TAPP repair to anterior open repair. The mean operation time was 109 minutes (range, 40–132) for unilateral hernia repair. Scrotal seroma was diagnosed at the operation site in 5 patients. No patient had operation-related orchitis, testicle edema, trocar site infection, or chronic pain during follow-up.* Conclusions.* The use of Polysoft mesh for TAPP inguinal hernia repair does not seem to adversely affect the quality of repair. The use of this mesh is therefore feasible and safe and may reduce postoperative pain.

## 1. Introduction

Prosthetic mesh has recently been used in the operative management of inguinal hernia and has been shown to significantly reduce recurrence as compared with traditional anterior hernia repair [1]. Although recurrence rates remain the most important outcome parameter, other variables, such as postoperative pain and discomfort, have attracted more attention as interest shifts to the postoperative quality of life. Recently, discussions on inguinal hernia repair focus more on chronic pain, rather than the rate of recurrence. The number of studies reporting high incidences of postoperative chronic pain after open anterior mesh repair is increasing [2, 3].

Pélissier reported that the preperitoneal placement of Polysoft mesh (C.R. Bard, Inc., Puerto Rico, USA) via an open anterior approach reduces postoperative disabling pain [4–6]. Preperitoneal placement of the mesh has the advantage of using the intra-abdominal pressure to push the mesh against the overlying fascia in a more natural type of repair and decreases postoperative chronic pain because it prevents contact with the inguinal sensory nerves (ilioinguinal, iliohypogastric, and genital branch of the genitofemoral nerve) running in the inguinal canal [7–9]. Polysoft mesh is a moderate-pore polypropylene mesh with a memory-ring consisting of polyester. The memory-ring offers easy deployment of the patch in the preperitoneal space during open anterior mesh repair.

Developments in laparoscopic techniques to repair inguinal hernias using polypropylene flat mesh have led to valuable options for the management of inguinal hernias [10–13]. Several studies have shown that laparoscopic repair offers the advantage of minimally invasive surgery to the patient [1, 14]. Laparoscopic repair is associated with less postoperative pain, prompter return to normal activities, and less chronic pain than classic open, tension-free, mesh hernia repair. Currently, most laparoscopic repairs of inguinal hernias are performed by placement of a mesh into the preperitoneal space. However, the use of Polysoft mesh for transabdominal preperitoneal (TAPP) hernia repair has not been reported previously. We report the results of a study evaluating the feasibility, safety, and effectiveness of TAPP inguinal hernia repair using Polysoft mesh.

## 2. Patients and Methods

### 2.1. Patients

From April 2010 through March 2013, we performed TAPP inguinal hernia repair using Polysoft mesh in 67 patients. All patients were 40 years or older and had a preoperative diagnosis of inguinal direct, indirect, femoral, or mixed hernia in the inguinal region. Patients with a history of lower abdominal or pelvic surgery were excluded. Recurrent inguinal hernias after nonpreperitoneal mesh repair and bilateral hernias were included in the analysis. Three different senior resident surgeons performed TAPP repair under the instruction of a specialist surgeon. If laparoscopic procedures could not create an adequate preperitoneal space because of technical reasons, conversion to conventional open anterior hernia repair was performed. Intraoperatively, we evaluated the type and size of the hernia and recorded the size of the Polysoft mesh used, that is, medium-size mesh (14 × 7.5 cm) or large-size mesh (16 × 9.5 cm). This retrospective study was carried out in accordance with the principles embodied in the Declaration of Helsinki, 2013. Informed consent for the study was obtained from all patients.

### 2.2. Surgical Technique

**Figure 1:**
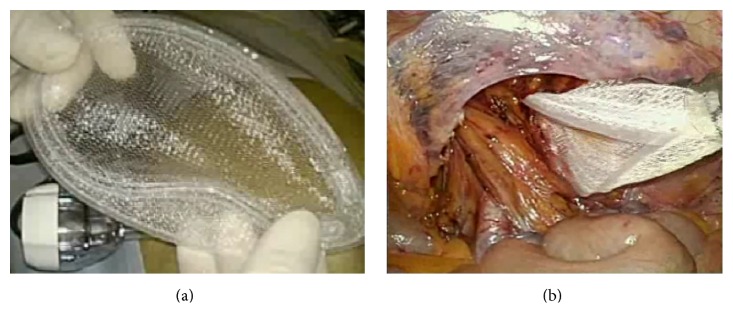
Polysoft mesh with the memory-ring (a). The mesh can be inserted into the abdominal cavity via a 12 mm port. Turning down the mesh in a dog-ear fashion using the interruption of the memory-ring (b).

**Figure 2:**
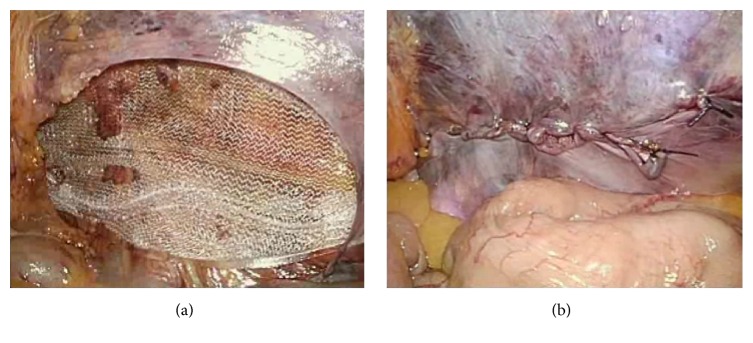
Laparoscopic view of Polysoft mesh in the preperitoneal space, covering the myopectineum of Fruchaud (a). The peritoneum is then closed with continuous sutures (b).

Under general anesthesia, the supine patient was placed on the operating table with the arms to the side and in a 10° to 20° Trendelenburg position. The television monitor was placed at the foot of the table. A 12 mm port was placed in the umbilical ring for laparoscopy and for CO_2_ pneumoperitoneum up to pressure of 10 mm Hg; two 5 mm working ports were placed, one in the right side and the other in the left side of the abdomen. The hernia was identified, and a peritoneal incision was made from the iliac tubercle extending medially to the umbilical ligament. The peritoneum and preperitoneal contents were bluntly dissected from the spermatic cord and vessels. The hernia sac was reduced meticulously, carefully preserving the epigastric vessels and vas deferens. The pubic tubercle was then clearly defined. Polysoft mesh was inserted into the abdominal cavity via the 12 mm port (Figures 1(a) and 1(b)). The size of Polysoft mesh should be adapted to the size of the hernia defect, so as to provide sufficient overlapping. On placing the mesh, it is important to expand the mesh medially into the preperitoneal space. At the lateral side of the internal ring, the mesh has to be adequately spread so that the entire myopectineum is covered, especially in patients with indirect hernias (Figure 2(a)). The mesh was fixed medially on the pubic tubercle and Cooper's ligament and along the superior margin of the prepared space away from the epigastric vessels, using 5 mm fixation devices for laparoscopic hernia repair (Covidien). No fixation devices were used at the inferior margin of the mesh to avoid nerve entrapment and vascular injury. The peritoneum was then closed with continuous 3-0 absorbable sutures (Figure 2(b)).

### 2.3. Evaluation

All patients were followed up on an outpatient basis 14 days after the operation to detect early complications such as scrotal seroma, hematoma, infection, testicle edema, and orchitis and to assess the patient's satisfaction with the procedure. The operation time and the duration of the postoperative hospital stay were recorded for every patient. To evaluate recurrence and chronic pain, patients were routinely assessed as outpatients or were contacted by telephone 3, 6, and 12 months postoperatively.

Activities were not limited by chronic pain in any patient, and all patients resumed their usual work or preoperative daily activities within 3 months. Postoperative chronic pain was classified according to Cunningham et al.'s definition [15]: mild, occasional pain or discomfort that did not limit activity; moderate pain preventing return to normal preoperative activities; and severe pain that incapacitated the patient at frequent intervals or interfered with daily activities.

## 3. Results

A total of 67 patients (62 men and 5 women; median age, 58 years; range, 45 to 76) who underwent TAPP hernia repair using Polysoft mesh were studied. There were a total 76 hernias, including 37 indirect inguinal hernias, 29 direct hernias, 1 femoral hernia, 1 pantaloon hernia (combined direct/indirect inguinal hernia), and 8 recurrent hernias. The eight recurrent hernias developed after open anterior hernia repair.

The median operation time for unilateral hernia repair was 109 minutes (range, 40–132). All patients underwent general anesthesia. During surgery, all pieces of Polysoft mesh placed were of adequate size. The mesh was not split in any patient and was able to be placed through a 12 mm trocar into the preperitoneal space. A medium-size Polysoft mesh (14 × 7.5 cm) was used in all patients. No large-size mesh (16 × 9.5 cm) was necessary because our Japanese patients had relatively small physiques.

TAPP hernia repair was not converted to traditional open anterior hernia repair in any patient. Furthermore, no respiratory, cardiac, or neurologic complication was noted intraoperatively. As for the postoperative hospital stay, 99% of the patients were discharged home within 3 days after surgery.

As for early postoperative complications, no patient had a scrotal hematoma that was drained under local anesthesia. Five patients were given a diagnosis of scrotal seroma developing at the site of operation. There were no cases of operation-related orchitis, testicle edema, or trocar site infection during follow-up.

Groin pain at postoperative 2-3 weeks was markedly lower than at postoperative 3 months. All patients suffering from postoperative pain with movement reported that this gradually reduced over time. No severe chronic pain was observed at postoperative 6 months.

The median follow-up was 32 months (range, 18–60 months). Early recurrence was clinically diagnosed 3 weeks after surgery in 1 patient. This patient immediately underwent open anterior hernia repair in our hospital.

## 4. Discussion

We designed this study to assess the feasibility and safety of TAPP hernia repair using memory-ring (Polysoft) mesh. Our results showed a low incidence of postoperative events. The technique of TAPP hernia repair using Polysoft mesh is feasible even for the repair of large, complex hernias, including pantaloon hernias and recurrent hernias, without any special difficulties.

Prosthetic mesh is now routinely used for inguinal hernia repair [16]. The low recurrence rates associated with mesh repair have shifted the attention of surgeons from recurrence to chronic pain after surgery. The chronic pain after onlay (over the floor of the inguinal canal) mesh placement has been attributed to fibrosis around the mesh. The fibrosis induced by the placement of onlay mesh at sites transversed by major inguinal sensory nerves (ilioinguinal, iliohypogastric, and genital branch of genitofemoral nerves) causes pain due to strong fixation of the mesh to the region around the inguinal canal [7]. Chronic pain has been classified into two types: nociceptive pain caused by tissue injury or an inflammatory reaction and neuropathic pain caused by direct nerve injury [17]. The continuous inflammation around the mesh may lead to nerve damage. The mesh, as a foreign body, induces a dense fibroblastic response that stimulates the formation of severe scar tissue. The increased size of the inflammatory area caused by the mesh may promote nerve adherence or abrasion that accounts for an increased risk of neuropathic pain. Placement of the mesh in the preperitoneal space offers the advantage of avoiding inflammatory response leading to the formation of severe scar tissue in the region of the inguinal sensory nerves and the spermatic cord [5–9]. This preperitoneal space is the same site used for laparoscopic approaches. Consequently, placement of the mesh in the preperitoneal space might have the advantage of decreasing postoperative chronic pain in both anterior and laparoscopic inguinal repair.

Pélissier reported the feasibility of transinguinal hernia repair in the preperitoneal space using Polysoft mesh, which sufficiently covered the myopectineum of Fruchaud [4–6]. Laparoscopic hernia repair using a Polysoft mesh offers several advantages over the use of a conventional flat mesh without a memory-ring. First, resident surgeons often find it difficult to spread a conventional flat mesh in laparoscopic surgery. A Polysoft mesh with a memory-ring is easier to spread completely without mesh deformity than a conventional flat mesh. Another advantage is that mesh migration is unlikely even if fixation of the mesh to the neighboring tissues is minimal, because a Polysoft mesh maintains its configuration. A potential disadvantage of Polysoft mesh might be complications resulting from breakage of the ring. However, ring breakage did not occur in our series.

With regard to postoperative pain, previous studies have reported that about 10% of patients experience severe chronic pain related impairment of everyday activities [2, 3, 18]. In two meta-analyses comparing open anterior repair with laparoscopic repair, laparoscopy seemed to be advantageous because it is associated with less postoperative pain, earlier recovery, and less absence from work [19, 20]. These advantages of laparoscopic repair may be explained by the preperitoneal location of the mesh far from the inguinal sensory nerves. Moreover, whether or not the inguinal canal is dissected is a main technical difference between open anterior hernia repair and laparoscopic hernia repair that might contribute to the differences in chronic severe pain rates. The preperitoneal placement of a Polysoft mesh with a memory-ring eliminates the need for extensive dissection. The risks of nerve entrapment by sutures or nerve irritation by the mesh are also reduced by using a preperitoneal mesh, because the preperitoneal placement of a Polysoft mesh does not require adequate fixation of the mesh by tacks and is not in contact with nerves running in the inguinal canal. Severe chronic pain did not occur in any of the patients of our series. We attempted to avoid chronic pain by limiting the number of tacks used (less than 3 tacks), and the use of Polysoft mesh might have also contributed to the good long-term results. The evaluation of chronic pain by telephone interview in this pilot study was not appropriate, because a patient would tend to be reluctant to answer negative outcome. A blind anonymous validated questionnaire with a physical examination would make a best collection of data.

In this study, only a few early postoperative complications (5%) occurred and they were benign; the four hematomas were superficial and did not require drainage. Hemorrhage did not occur. To evaluate recurrence, we reexamined the patients 3 months after operation. A telephone questionnaire was conducted for follow-up 12 months after operation. There was only 1 case of recurrence (supravesical hernia). Our recurrence rate of 4.3% (1 of 67 patients) lies within the range reported in the literature for laparoscopic inguinal hernia repair [11–13].

Because our study focused on the feasibility and safety of TAPP hernia repair using Polysoft mesh the follow-up period was not long enough to fully assess the long-term results of such repair. To date, however, our follow-up data suggest that the use of Polysoft mesh does not have any adverse effects on the quality of repair. Therefore, randomized clinical controlled trail of TAPP hernia repair with the use of Polysoft mesh versus other mesh is needed and planning to confirm the short- and long-term outcomes, including postoperative chronic pain.

## 5. Conclusion

Our results indicate that TAPP hernia repair using Polysoft mesh is a safe, easy, and minimally invasive procedure.

## References

[1] Butters M., Redecke J., Köninger J. (2007). Long-term results of a randomized clinical trial of Shouldice, Lichtenstein and transabdominal preperitoneal hernia repairs. *British Journal of Surgery*.

[2] Bay-Nielsen M., Perkins F. M., Kehlet H. (2001). Pain and functional impairment 1 year after inguinal herniorrhaphy: a nationwide questionnaire study. *Annals of Surgery*.

[3] Hawn M. T., Itani K. M., Giobbie-Hurder A., McCarthy M., Jonasson O., Neumayer L. A. (2006). Patient-reported outcomes after inguinal herniorrhaphy. *Surgery*.

[4] Pélissier E. P. (2006). Inguinal hernia: preperitoneal placement of a memory-ring patch by anterior approach. Preliminary experience. *Hernia*.

[5] Pélissier E. P., Blum D., Ngo P., Monek O. (2008). Transinguinal preperitoneal repair with the Polysoft patch: prospective evaluation of recurrence and chronic pain. *Hernia*.

[6] Pélissier E. P., Monek O., Blum D., Ngo P. (2007). The Polysoft patch: prospective evaluation of feasibility, postoperative pain and recovery. *Hernia*.

[7] Berrevoet F., Maes L., Reyntjens K., Rogiers X., Troisi R., De Hemptinne B. (2010). Transinguinal preperitoneal memory ring patch versus Lichtenstein repair for unilateral inguinal hernias. *Langenbeck's Archives of Surgery*.

[8] Berrevoet F., Sommeling C., De Gendt S., Breusegem C., de Hemptinne B. (2009). The preperitoneal memory-ring patch for inguinal hernia: a prospective multicentric feasibility study. *Hernia*.

[9] Maillart J. F., Vantournhoudt P., Piret-Gerard G., Farghadani H., Mauel E. (2011). Transinguinal preperitoneal groin hernia repair using a preperitoneal mesh preformed with a permanent memory ring: a good alternative to Lichtenstein's technique. *Hernia*.

[10] Moldovanu R., Pavy G. (2014). Laparoscopic Transabdominal Pre-Peritoneal (TAPP) procedure—step-by-step tips and tricks. *Chirurgia*.

[11] Sato H., Shimada M., Kurita N. (2012). The safety and usefulness of the single incision, transabdominal pre-peritoneal (tapp) laparoscopic technique for inguinal hernia. *Journal of Medical Investigation*.

[12] Tzovaras G., Symeonidis D., Koukoulis G. (2012). Long-term results after laparoscopic transabdominal preperitoneal (TAPP) inguinal hernia repair under spinal anesthesia. *Hernia*.

[13] Zacharoulis D., Fafoulakis F., Baloyiannis I. (2009). Laparoscopic transabdominal preperitoneal repair of inguinal hernia under spinal anesthesia: a pilot study. *American Journal of Surgery*.

[14] Liem M. S. L., van Duyn E. B., van der Graaf Y., van Vroonhoven T. J. (2003). Recurrences after conventional anterior and laparoscopic inguinal hernia repair: a randomized comparison. *Annals of Surgery*.

[15] Cunningham J., Temple W. J., Mitchell P., Nixon J. A., Preshaw R. M., Hagen N. A. (1996). Cooperative hernia study: pain in the postrepair patient. *Annals of Surgery*.

[16] Bittner R., Schwarz J. (2012). Inguinal hernia repair: current surgical techniques. *Langenbeck's Archives of Surgery*.

[17] Hakeem A., Shanmugam V. (2011). Current trends in the diagnosis and management of post-herniorraphy chronic groin pain. *World Journal of Gastrointestinal Surgery*.

[18] Poobalan A. S., Bruce J., Smith W. C., King P. M., Krukowski Z. H., Chambers W. A. (2003). A review of chronic pain after inguinal herniorrhaphy. *Clinical Journal of Pain*.

[19] Memon M. A., Cooper N. J., Memon B., Memon M. I., Abrams K. R. (2003). Meta-analysis of randomized clinical trials comparing open and laparoscopic inguinal hernia repair. *British Journal of Surgery*.

[20] Schmedt C. G., Sauerland S., Bittner R. (2005). Comparison of endoscopic procedures vs Lichtenstein and other open mesh techniques for inguinal hernia repair: a meta-analysis of randomized controlled trials. *Surgical Endoscopy and Other Interventional Techniques*.

